# Crystal-Plasticity-Finite-Element Modeling of the Quasi-Static and Dynamic Response of a Directionally Solidified Nickel-Base Superalloy

**DOI:** 10.3390/ma13132990

**Published:** 2020-07-05

**Authors:** Rafael Sancho, Javier Segurado, Borja Erice, María-Jesús Pérez-Martín, Francisco Gálvez

**Affiliations:** 1 Department of Materials Science, E.T.S.I Caminos, Canales y Puertos, Universidad Politécnica de Madrid, 28040 Madrid, Spain; javier.segurado@upm.es (J.S.); f.galvez@upm.es (F.G.); 2 IMDEA Materials Institute, C/Eric Kandel 2, Getafe, 28906 Madrid, Spain; 3 Department of Mechanics and Industrial Production, Mondragon Unibertsitatea, Loramendi 4, 20500 Mondragon, Spain; berice@mondragon.edu (B.E.); mjperez@mondragon.edu (M.-J.P.-M.); 4 IKERBASQUE, Basque Foundation for Science, 48013 Bilbao, Spain; 5 Structural Impact Laboratory (SIMLab), Department of Structural Engineering, Norwegian University of Science and Technology (NTNU), NO-7491 Trondheim, Norway; 6 Centre for Advanced Structural Analysis (CASA), NTNU, NO-7491 Trondheim, Norway

**Keywords:** crystal plasticity, nickel-base superalloy, oligocrystal, finite element method, high strain rates

## Abstract

The flow stress behaviour of a directionally solidified nickel-base superalloy, MAR-M247, is presented through the combination of experiments and crystal-plasticity simulations. The experimental campaign encompassed quasi-static and dynamic testing in the parallel and perpendicular orientation with respect to the columnar grains. The material showed low strain-rate sensitivity in all cases. Virtual samples were generated with DREAM3d and each grain orientation was established according to the DS nature of the alloy. The elasto-visco-plastic response of each crystal is given by phenomenological-base equations, considering the dislocation–dislocation interactions among different slip systems. The hardening-function constants and the strain-rate sensitivity parameter were fitted with the information from tests parallel to the grain-growth direction and the model was able to predict with accuracy the experimental response in the perpendicular direction, confirming the suitability of the model to be used as a tool for virtual testing. Simulations also revealed that in oligocrystalline structures of this type, the yield-strength value is controlled by the grains with higher Schmid factor, while this influence decreases when plastic strain increases. Moreover, the analysis of the micro-fields confirmed that grains perpendicular to the loading axis are prone to nucleate cavities since the stresses in these regions can be twice the external applied stress.

## 1. Introduction

Turbine blades are components that work under a wide range of operating conditions, leading to a combination of thermo-mechanical fatigue, creep and impact loads under a corrosive environment. Iron, cobalt and nickel-base superalloys are the three major classes of materials that are able to keep the required mechanical performance at high temperatures [1], nickel-base superalloys being the most widely used in aerospace industry. The reason for the good mechanical properties of the previously-mentioned alloys at high temperatures is grounded in their microstructure, a face-centred-cubic (FCC) matrix, called γ phase, strengthened with a high volume fraction of $L1_{2}$ ordered $Ni_{3}\left(Ti,Al\right)$ precipitates, called $\gamma^{{}^{\prime }}$ phase. The thermal softening of the matrix is offset by the thermal hardening of the precipitates, keeping almost constant the yield strength evolution of the alloy or even getting higher values at some temperature ranges [2,3,4]. For the last few decades, the performance of these alloys has been continuously improved not only by adjusting the amount of alloying elements and the time/temperature conditions of the heat treatments, but also by changing the processing technique to create polycrystalline, directionally solidified (DS) or single crystal (SX) components. The polycrystalline alloys are the cheapest and easiest option for manufacturing, but the high number of grain boundaries has a negative effect on the mechanical performance. Grain-boundary sliding [5] and Coble creep [6] promote permanent deformation of the components at $T>0.4T_{m}$, decreasing the creep resistance, while stress concentration facilitates cavity nucleation in grain boundaries perpendicularly to the loading direction [7]. Therefore, in the aerospace industry, DS alloys are of great interest because of the pair price/performance, being that they are cheaper and easier to manufacture than SXs and with superior mechanical properties than polycrystals. Their microstructure is characterised by columnar grains, aligned with the principal-stress axis (-z- axis by convention), that grow in the preferred crystallographic orientation <001> [8,9], leading to considerable anisotropy when the loading changes from the solidification direction to the perpendicular one. Different approaches have been used to model the constitutive response of heterogeneous structures of this type, going from transversally isotropic viscoplastic models [10,11], through the use of self-consistent schemes [12,13], to the crystal-plasticity-finite-element method (CPFEM) [14]. Transversally isotropic models are of interest when the computational cost is an issue, i.e., simulating a full structural component, but present three main drawbacks: (1) the assumption of one plane of isotropy is not true for a small numbers of grains in the cross-section, (2) strain and stress-fields’ concentrations among grains are not replicated and (3) mechanical tests in at least three different directions are needed to fit the constants. These disadvantages can be overcome by representing explicitly the oligocrystalline microstructure and with the use of CP, since it takes into account the main deformation mechanisms, the shape and orientation of each grain. In previous studies, CPFEM models have been used to investigate the fatigue behaviour of DS Ni-base alloys [14], the stress and strain fields close to a crack tip [15] or the strain-field localisation in oligocrystalline specimens with the aim of analysing fatigue-crack nucleation [16]. Focusing the attention on the dynamic loading—only a few studies can be found which model this regime. However, these studies are focused on standard polycrystalline microstructures and rely on more simple models, such as Visco plastic self consistent (VPSC) [17], or are applied to other types of alloys, such as Ti ones [18,19]. No work has been found, to the authors knowledge, which analyses the mechanical response of any DS superalloy at high strain rates using CPFE models.

In the case of DS specimens or parts, containing only a few large grains, this technique offers several advantages with respect to macroscopic approaches or mean field models. It allows the researchers to accurately model the macroscopic response of a sample accounting for the texture and grain morphology with a reduce number of parameters. Additionally, the simulation provides information about field distribution within the grains, which can be used to understand the microscopic deformation mechanisms and study the effect of microstructure in the failure of the material.

The present work aims at covering this gap and presents a study, using both experimental and modelling techniques, of the dynamic and anistropic behaviour of MAR-M247 DS alloy. The experimental part encompasses quasi-static ($\dot{\epsilon }$ = 2.5 × 10${}^{-4}$ s${}^{-1}$) and high-strain-rate ($\dot{\epsilon }$ = 150 s${}^{-1}$, $\dot{\epsilon }$ = 500 s${}^{-1}$ ) tests in the parallel and perpendicular direction to grain-growth direction, which adds information about the flow stress and anisotropic behaviour of the MAR-M247 alloy at different strain rates to the already published literature that presents its performance under thermo-mechanical fatigue [20,21], creep fatigue [22] or the influence of the temperature, grain size and alloying elements on engineering parameters such as the yield strength or the ultimate tensile strength [23,24].

Then, a computational micromechanics approach is used to model the experiments. The numerical strategy presented in this work, based on CPFEM, accounts for the explicit representation of the grain characteristics (size, aspect ratio and orientation) along the specimen gauge length based on the experimentally measured grain-size distribution. The response of each grain is modelled by using a standard visco-plastic approach [25], in which viscous slip is described with a power law, and the evolution of the critical resolved shear stress (CRSS) is considered through a phenomenological hardening model with latent hardening coefficients taken from lower-scale simulations [26]. The parameters of the hardening law and the viscous response are adjusted by only using the quasi-static and dynamic experimental data in the grain-growth direction (<001>), while the results in the perpendicular direction are employed for checking the prediction capabilities of the numerical strategy. Finally, the simulations are used to analyse the stress micro-fields for each configuration, showing an important stress concentration on grain boundaries for 90${}^{\circ }$ samples, which may promote cracks and/or cavities nucleation.

## 2. Materials and Methods

### 2.1. Material Description

The studied material was the MAR-M247 DS alloy, a casting nickel-base superalloy that was developed in the early 1970s [27]. Its microstructure consists of a solid-solution-strengthened matrix (γ phase) with around 60% of ${\mathrm{Ni}}_{3}\left(\mathrm{Ti},\mathrm{Al}\right)$ precipitates ($\gamma^{{}^{\prime }}$ phase). The γ phase has an FCC lattice while the precipitates have an $L1_{2}$ ordered structure. This structure is similar to the FCC one, but the corner and the face-centred sites are occupied by different atoms; e.g., Al atoms in the corners and Ni atoms in the faces sites for intermetallic compound ${\mathrm{Ni}}_{3}\mathrm{Al}$. The nominal chemical composition, given by the material supplier GKN Aerospace, can be seen in Table 1.

**Figure 1:**
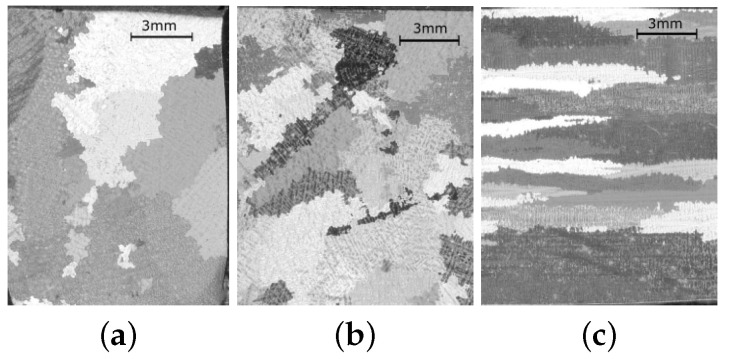
Microstructure of the MAR-M247DS alloy. The images (**a**,**b**) show the grains of the cross-section perpendicular to grain-growth direction, while the image (**c**) is in the parallel direction.

The bulk material was cut into three different prismatic samples of size 12 × 15 × 12 mm to study the microstructure by optical microscopy before testing. The samples were polished up to 1μm and etched with a solution of 1 mL of $H_{2}O_{2}$ + 2 mL of HCl + 3 mL of $H_{2}O$ for one minute to reveal the microstructure, proving the existence of columnar grains with sizes in the range of several millimetres (see Figure 1). The images were obtained by using an Optech LFZ zoom stereomicroscope equipped with a ProgRes digital camera.

**Figure 2:**
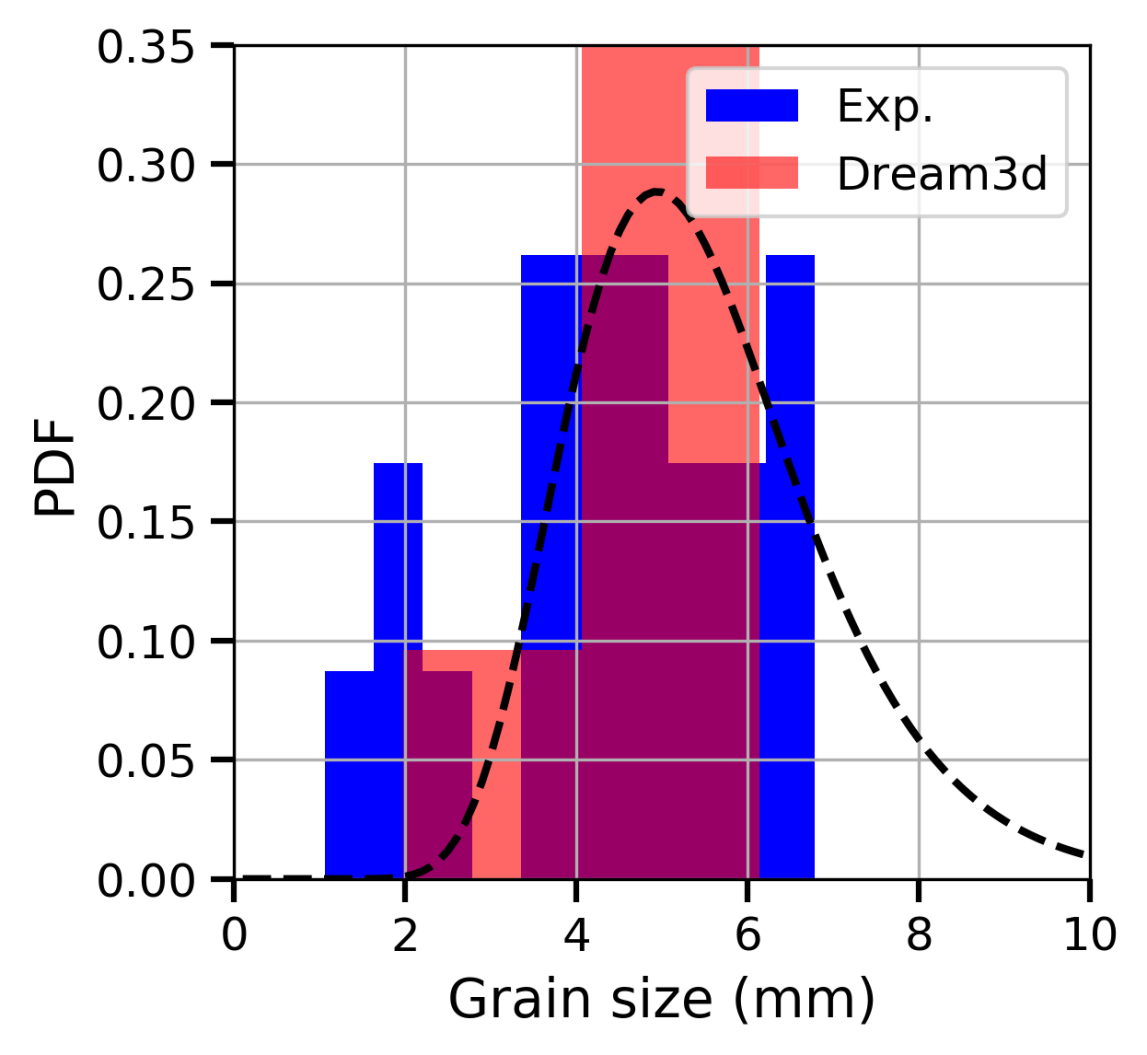
Experimental grain-size distribution (blue histogram) and the log-normal fitting (dashed line) of the alloy. The mean size is 5.5 mm. The virtual grain-size distribution generated with Dream3D [29] is also represented.

Size and shape distributions were analysed from the microscopic images obtained in the longitudinal and transverse directions through the software IMAGE J [28]. Each grain was adjusted to an ellipse with the same area, and the characteristic length was obtained as $0.5\left(a+b\right)$, where *a* and *b* are the major and minor axis respectively. The analysis of 25 grains showed that the size may vary from one to seven millimetres in the cross-section perpendicular to the grain-growth direction (see Figure 2). The data were fitted with a log-normal distribution, getting the values $d_{g}=1.67$ ln(mm), $d_{SD}=0.073$ ln(mm), which are, respectively, the mean and standard deviation of the variables’ natural logarithm. A mean grain size of 5.5 mm was obtained.

### 2.2. Mechanical Characterisation

**Figure 3:**
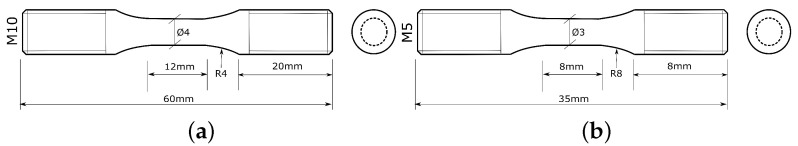
Schematic representation of the axisymmetric samples. Quasi-static and 500 $s^{-1}$ dynamic tests were performed with the sample on the left (**a**) and the 150 $s^{-1}$ dynamic tests with the sample depicted in (**b**) .

The inelastic behaviour of the alloy was characterised by means of quasi-static and high-strain-rate tests on axisymmetric samples obtained parallel (0${}^{\circ }$) and perpendicularly (90${}^{\circ }$) to the grain-growth direction (-z- axis by convention). The specimens were machined with the geometry and dimensions detailed in Figure 3. The sample (**a**) was used in the tests performed at 1.5 ×$10^{-4}$ s${}^{-1}$ and 500 $s^{-1}$, while the sample (**b**) was employed in the tests carried out at a strain rate of 150 $s^{-1}$. The latter had a different geometry due to limitations of the set-up used for conducting these particular experiments.

#### 2.2.1. Quasi-Static Tensile Tests

**Figure 4:**
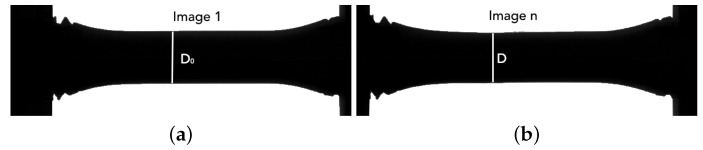
Example of the edge tracing technique used for monitoring the minimum sample diameter during testing. *Image 1* (**a**) corresponds to the frame when the test starts and *Image n* (**b**) corresponds to the frame just before to failure.

Four quasi-static tensile tests were carried out on samples oriented at 0${}^{\circ }$ and 90${}^{\circ }$ at room temperature. The tests were performed in an INSTRON servo-hydraulic universal testing machine at an engineering strain rate of $\dot{e}$ = 1.5 ×$10^{-4}$ s${}^{-1}$. Each test was recorded with a video camera, using a frame rate of two images per second, and the edge-tracing technique, implemented in the software eCorr [30], was used to monitor the evolution of the cross-section diameter *D* of the specimens (Figure 4). The true stress σ and the logarithmic strain ϵ were then calculated as:

(1) $$\sigma =\frac{4F}{\pi D^{2}};\;\epsilon =2ln\left(\frac{D_{0}}{D}\right)$$

where *F* is the force and $D_{0}$ is the initial cross-section diameter.

#### 2.2.2. Dynamic Tensile Tests

**Figure 5:**
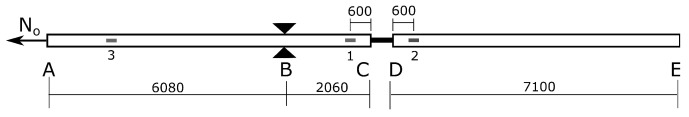
Dimensions of the split Hopkinson tension bar (SHTB) set-up. The strain gauges are in the positions 1, 2 and 3. Image adapted from [33]. For more information about this SHTB apparatus, consult the same citation.

Dynamic tensile tests of axisymmetric smooth specimens were carried out in a split Hopkinson tension bar (SHTB) [31]. In this experimental technique, the sample is fastened between two bars that are instrumented with strain gauges (see Figure 5). An incident tensile stress pulse is generated in the input bar and when it reaches the bar/specimen interface, part of the wave is transmitted to the second bar (output bar) and the rest is reflected back towards the input bar. By measuring the incident $\epsilon_{i}$, reflected $\epsilon_{r}$ and transmitted $\epsilon_{t}$ strain signals, and applying the theory of wave propagation in 1D elastic media, the force *F* and the displacement Δl applied to the sample are calculated with the equations [32]:

(2) $$\begin{array}{c} F=E_{b}A_{b}\epsilon_{t}=E_{b}A_{b}\left(\epsilon_{i}+\epsilon_{r}\right)\\ \Delta l=-\int_{0}^{t}2c_{0}\epsilon_{r}dt \end{array}$$

where $E_{b}$ is the Young’s modulus, $A_{b}$ the cross-sectional area and $c_{0}$ the wave velocity of the bars.

In this experimental campaign, two different set-ups were used. In the first one [33] that is depicted in Figure 5 and that was used for the tests at $\dot{e}$ = 150 s${}^{-1}$, the incident bar is clamped in the point B. A homogeneous stress profile $\sigma_{0}$ is generated in the bar segment $\bar{AB}$ through prestressing it with a force $N_{0}$, which in turn, creates an incident tensile stress wave with a magnitude equal to 0.5 $\sigma_{0}$ when the clamp is released. The second SHTB system, which was used for the tests at $\dot{e}$ = 500 s${}^{-1}$, is similar to the one sketched in the present section but with some differences, such as the dimensions, the materials of the bars and the way to generate the pulse. The technical details of the system can be found in [34]. In this case, the incident stress wave is generated by impacting a 0.4 m-length cylindrical projectile inside a 3 m-length gas gun, which is launched with compressed air, against a flange connected to the end of the input bar.

Finally, the stress–strain relationship was computed through the use of the true stress and logarithmic strain defined in Equation (1). The force was obtained from the strain-gauge signals, while the cross-section-diameter evolution was monitored by a Phantom V2511 high-speed camera and the edge tracing technique implemented in eCorr [30]. The camera was set up with a resolution of 640 × 208 px and a frame rate of 150,000 fps.

### 2.3. Numerical Modelling

#### 2.3.1. Crystal Plasticity Model

The crystal plasticity model used to simulate the behaviour of the alloy was formulated and implemented in ABAQUS FEA code by the strategy followed in [25]. The model assumes that the deformation gradient F can be decomposed multiplicatively into elastic $F^{e}$ and plastic $F^{p}$ parts according to [35]:

(3) $$F=F^{e}F^{p}$$

Using the definition of the velocity gradient $L\equiv \nabla_{x}v=\dot{F}F^{-1}$, the expression (3) leads to the additive decomposition of L as:

(4) $$L=L^{e}+F^{e}L^{p}F^{e^{-1}}$$

Since plastic deformation in a crystal takes place along different slip systems α, the plastic deformation gradient is defined as:

(5) $$L^{p}=\sum_{\alpha }\dot{\gamma^{\alpha }}\left(s^{\alpha }⊗m^{\alpha }\right)$$

where $\dot{\gamma^{\alpha }}$ is the plastic slip rate on the slip system α and $s^{\alpha }$ and $m^{\alpha }$ stand, respectively, for the unit vectors in the slip direction and normal to the slip plane in the reference configuration. In nickel-base superalloys, deformation takes place due to the contributions of octahedral (FCC matrix + $L1_{2}$ precipitates) and cube ($L1_{2}$ precipitates) slip systems [36,37]. However, the importance of the different deformation mechanisms depends on temperature [38]. It is well accepted that at low temperatures, in Ni-base superalloys, most dislocations lie in the matrix and those that shear the precipitates move in the $\gamma^{{}^{\prime }}$ octahedral planes too. It is not until higher temperatures, above 600 K according to [36], when cube slip in $\gamma^{{}^{\prime }}$ phase activates, pinning mobile dislocations occur and the flow strength of the alloy increases. In the present study, the material behaviour is described as a solid with 12 {111}<110> slip systems since only room temperature is considered. This approximation has been used and considered accurate enough by other authors [7,16] and implies that the CRSS and hardening behaviour of the octahedral systems accounts for the strengthening effect of the $\gamma^{{}^{\prime }}$ precipitates.

The crystal is assumed to behave as an elasto-viscoplastic solid in which the plastic-slip rate follow the power law [39]:

(6) $${\dot{\gamma }}^{\alpha }={\dot{\gamma }}_{0}{\left(\frac{\left|\tau^{\alpha }\right|}{g^{\alpha }}\right)}^{1/m}sign\left(\tau^{\alpha }\right)$$

where ${\dot{\gamma }}_{0}$ is the reference shear-strain rate, $g^{\alpha }$ the critical shear stress, $\tau^{\alpha }$ the resolved shear stress and *m* the rate-sensitivity exponent. The resolved shear stress τ on the slip system α is obtained by the projection of the second Piola–Kirchhoff stress tensor S on the corresponding slip system as:

(7) $$\tau^{\alpha }=S:s^{\alpha }⊗m^{\alpha }$$

S being the double inner product between the fourth order elastic stiffness tensor *C* and Green–Lagrange strain tensor $E^{e}$:

(8) $$S=C:E^{e}$$

The evolution of the critical resolved shear stress (CRSS), $g^{\alpha }$, for a given slip system α, is expressed as:

(9) $${\dot{g}}^{\alpha }=\sum_{\beta }Q_{\alpha \beta }h\left(\Gamma \right){\dot{\gamma }}_{\beta }$$

where $Q_{\alpha \beta }$ is a 12 × 12 symmetric matrix [40] that describes, with six independent constants ($q_{1}\ldots q_{6}$) in the case of FCC materials, the strength of the different interactions between pairs of slip systems. The values of the self and latent-hardening coefficients $Q_{\alpha \beta }$ (Table 2) have been adapted from the values obtained by using dislocation dynamics simulations in a FCC metal [26]. The first three terms ($q_{1},q_{2}$ and $q_{3}$) account, respectively, for self-interaction of dislocations in the same slip system, interaction of coplanar dislocations and interaction between collinear dislocations. The remaining terms ($q_{4},q_{5}$ and $q_{6}$) have to do with dislocation junctions (Hirth lock, glissile junction and Lomer–Cottrell lock) [40]. Finally, the hardening law takes the form [39,41]:

(10) $$h\left(\Gamma \right)=h_{0}sech^{2}\left(\frac{h_{0}\Gamma }{\tau_{s}-\tau_{0}}\right)$$

where $h_{0}$ is the initial hardening modulus and $\tau_{0}$ and $\tau_{s}$ stand for the initial and the saturation values of the CRSS, respectively. The values of these three coefficients were fitted with the experimental data of quasi-static tensile tests in the grain-growth direction. Γ is the accumulated strain on all the slip systems formulated as:

(11) $$\Gamma =\sum_{\alpha }\left|{\dot{\gamma }}^{\alpha }\right|dt$$

The model does not consider the effect of temperature because all mechanical tests were performed at 300 K and the effect of adiabatic heating can be disregarded since the yield strength of the alloy is temperature independent until 800 K [23,42].

#### 2.3.2. Numerical Set-Up

**Figure 6:**
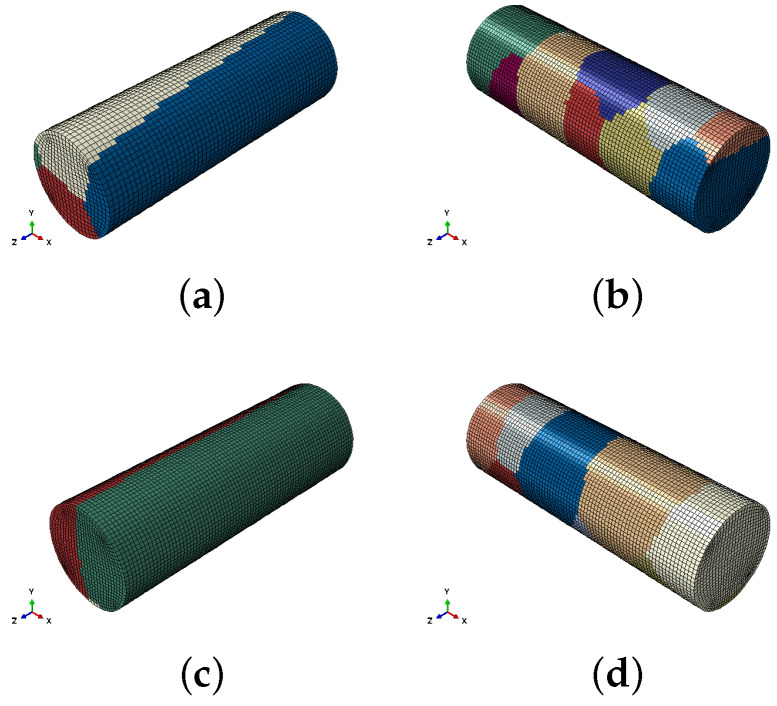
Illustration of the cylindrical FE models used to simulate the gauge length of the specimens. Images (**a**,**c**) depict models with grains parallel to grain-growth direction (0${}^{\circ }$), while figures (**b**,**d**) depict models where the axial load is perpendicular to grain-growth direction (90${}^{\circ }$). Each colour represents a different grain.

The specimens tested in this study are oligocrystals and for that reason the mechanical response was obtained by means of simulating the whole gauge length (Figure 6) instead of using a representative volume element (RVE). The cylindrical FE models were generated through a Python script that assigns grain IDs to each element of a structured mesh of the cylindrical gauge, assuming that is embedded in a larger cubic volume (24 × 24 × 24 mm) that contains a grain distribution representative of the experimental one (see Figure 1). The microstructure of this cube was generated with Dream3D [29] through the employment of ellipsoidal grains characterised by an aspect ratio of 18:1 in the grain growth direction, a mean equivalent-sphere-diameter of 9.2 mm and a standard deviation equal to 1.04 mm.

**Figure 7:**
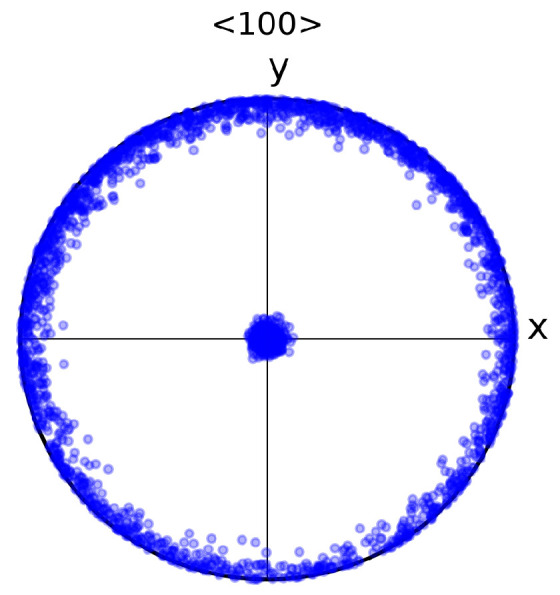
Stereographic projection of the crystallographic orientation of 1000 grains defined by the Euler-angles normal distribution $\phi_{1}$ = 20 ${}^{\circ }$± 90${}^{\circ }$, Φ = 0${}^{\circ }$± 4${}^{\circ }$, $\phi_{2}$ = 32${}^{\circ }$± 20${}^{\circ }$.

The crystallographic orientation (see Figure 7) was generated by assuming a fibre texture in which all grains have a <100> direction oriented near the z axis—the perpendicular directions being quasi-randomly oriented. This texture corresponds to the typical texture obtained in DS FCC alloys [8,9,14]. The particular Euler angles (using the Bunge convention) used to generate the fibre texture are $\phi_{1}$ = 20${}^{\circ }$± 90${}^{\circ }$, Φ = 0${}^{\circ }$± 4${}^{\circ }$, $\phi_{2}$ = 32${}^{\circ }$± 20${}^{\circ }$.

The specimens were discretised with 0.15 mm-size eight-node fully-integrated hexahedral elements (C3D8). The 3 mm-diameter samples were reproduced with 41,400-elements mesh and the 4 mm-diameter samples with 55,040-elements mesh. The prescribed displacement was the same (1.5 mm) in all tests and the total time of the step was adjusted to get the three different strain rates: 1.5 × 10${}^{-4}$ s${}^{-1}$, 150 s${}^{-1}$ and 500 $s^{-1}$. The quasi-static problem was solved with the Abaqus static solver while the dynamic implicit version was used for the high-strain-rate tests, where setting two time increments allowed the achievement of stress-waves propagation and time-cost-effective simulations alike. During the elastic regime, a time increment 10 times larger than the Courant number was chosen, whereas a time increment 100 times larger than the Courant number was used in the inelastic regime once the stress equilibrium was reached.

**Figure 8:**
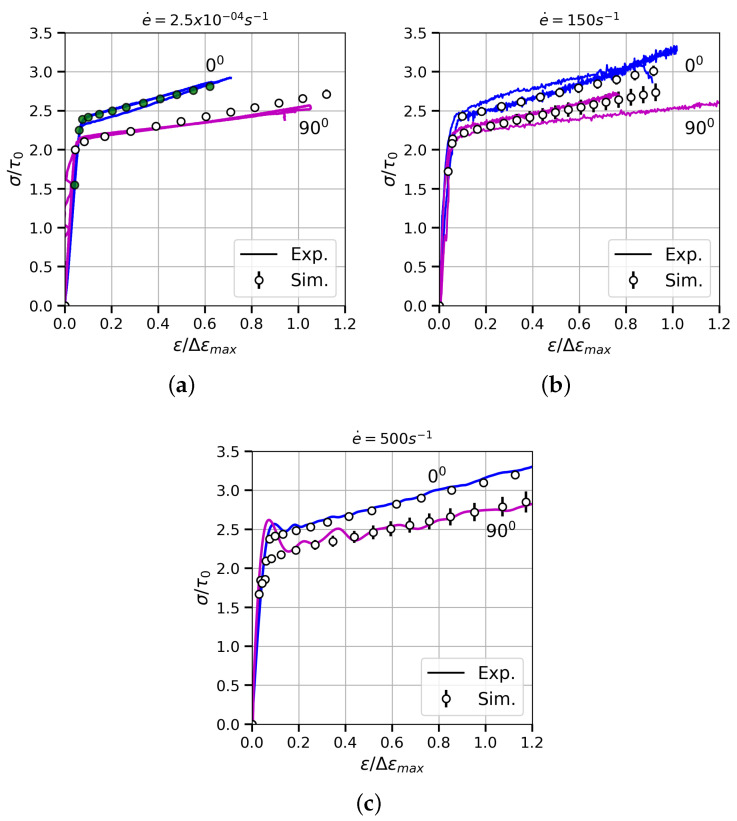
Experimental (lines) and numerical (markers) true stress–strain curves for MAR-M247DS alloy at room temperature under quasi-static (**a**) and dynamic (**b**,**c**) regimes and different loading directions. The green markers in the plot (**a**) are related to the fitting of the hardening law.

The set of parameters $h_{0}$, $\tau_{0}$, $\tau_{s}$ that describes the hardening behaviour were obtained by inverse analyses through only fitting the experimental data from the two tests performed under quasi-static conditions and at 0${}^{\circ }$ direction (green circles in Figure 8). To that end, a model with 5580 elements was used. Moreover, the yield-strength evolution at the different strain rates in the grain-growth direction allowed the determination of the rate-sensitivity parameter *m*. All these values, together with the elastic constants of the stiffness tensor obtained from [43], are gathered in Table 2.

## 3. Results and Discussion

Figure 8 exhibits the experimental true stress–strain curves (lines) of the studied alloy under different loading rates. It is important to note that both axes, horizontal and vertical, are non-dimensionalised with respect to $\Delta \epsilon_{max}$ and $\tau_{0}$, respectively, for reasons of confidentiality. In each graph there are two families of tests that belong to the samples obtained parallelly (0${}^{\circ }$) and perpendicularly (90${}^{\circ }$) to the grain-growth direction. The Young’s modulus *E*, the yield strength $\sigma_{0.2}$ and the hardening behaviour are modified by the sample orientation with the same trend for all the strain rates. Regarding the elastic behaviour, the Young’s modulus varies by 33% depending on whether the samples are parallel ($36.72\tau_{0}/\Delta \epsilon_{max}$) or perpendicular ($48.57\tau_{0}/\Delta \epsilon_{max}$) to the solidification direction. The yield strength, calculated as the 0.2 offset yield point, also changes. The larger values correspond to the 0${}^{\circ }$ orientation ($\sigma_{0.2}=2.37\tau_{0}$), while the performance in the transverse direction decreases up to quantities close to $2.11\tau_{0}$. Moreover, a variation on the hardening is observed when comparing the two directions. As expected, the best mechanical properties were obtained in the grain-growth direction: a lower Young’s modulus ensures high thermal-fatigue resistance because of the low thermal strains and a stronger work hardening. It is important to note that there is some dispersion in the results since oligocrystals were tested. These differences among curves are more noticeable in the case of the specimens oriented at 90${}^{\circ }$ due to the randomly oriented crystals.

As it was mentioned previously, Ni-base superalloys have an FCC matrix hardened with a high volume fraction of precipitates (long-range obstacles). Therefore, the amount CRSS associated with obstacles that can be overcome by thermal activation is very low, making these alloys almost rate and temperature insensitive [44]. This theoretical approach is confirmed by the experimental data shown in the current investigation (comparison among the subplots in Figure 8).

The simulation strategy presented in this work was able to faithfully predict (see Figure 8) the constitutive behaviour of the 90${}^{\circ }$-oriented DS Ni-base superalloy in all strain rates by using the model parameters fitted with the grain-growth-direction experimental data. The plots show the mean values (markers) with the standard deviation obtained from the numerical campaign, which consisted of two simulations per orientation for all strain rates. It is important to note that each simulation has a different grain structure (Figure 6) and crystal orientation to try to imitate the process of machining samples from the bulk material.

**Figure 9:**
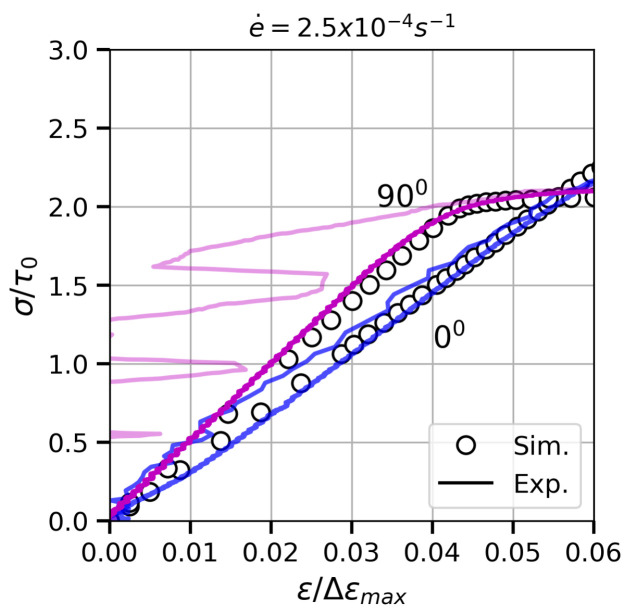
Elastic part of the experimental (coloured lines) and numerical (markers) true stress–strain curves for the quasi-static tests.

The <100>-fibre-texture hypothesis assumed to generate the models was confirmed, according to the equivalence between experimental and numerical results in both orientations in the elastic regime (Figure 9). Furthermore, the suitability of the elastic stiffness tensor calculated in [43] is also demonstrated. Note that one of the tests at 90${}^{\circ }$ (soft colour) showed high noise-to-signal ratio in the elastic regime.

**Figure 10:**
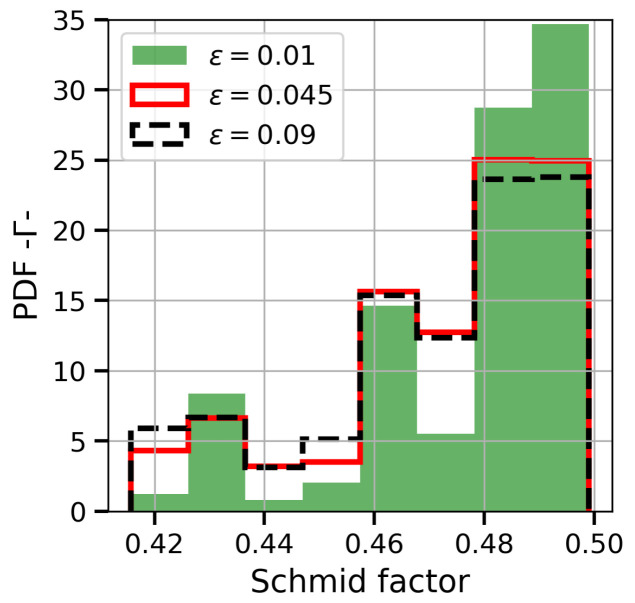
Probability density function of the total plastic shear strain Γ depending on the grain Schmid factor for the samples oriented perpendicularly. The data were obtained for three different levels of applied true strain: at the beginning of plastic deformation -0.01-, in the middle of the test -0.045- and in the final stages -0.09-.

The difference in the yield strength $\sigma_{0.2}$ between the two sample configurations is captured by the different maximum Schmid factors $m_{max}$ (the higher the $m_{max}$, the lower the external applied stress to move dislocations). At 0${}^{\circ }$, the texture is highly oriented in <001> and the $m_{max}$ of most of crystals is close to 0.408. On the other hand, the grain orientations of 90${}^{\circ }$ specimens are more random and the maximum Schmid factor may take values between 0.408 and 0.50. For example, in the perpendicular simulation, on average, 21% of grains had maximum Schimd factors below 0.44 and 40% above 0.47. Moreover, in structures of this type, the grains with higher $m_{max}$ tend to accommodate more plastic deformation in the beginning of yielding, as can bee seen in Figure 10.

Since the CRSS depends implicitly on the total amount of plastic strain through the hardening function (Equations (9) and (10)), the grains with higher plastic strain harden faster, and therefore, there is a certain moment at which the grains with lower $m_{max}$ start to offer less resistance to yielding. The aforementioned graph reveals how the probability density function PDF of plastic deformation rises for grains with low $m_{max}$ and drops for large $m_{max}$ when the total true strain growths.

**Figure 11:**
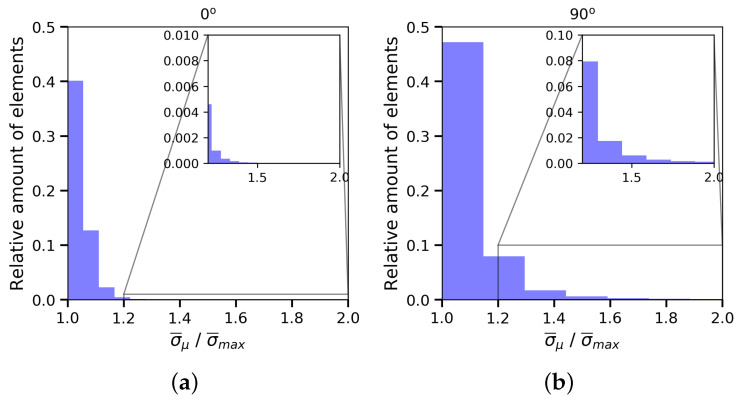
Histogram to represent, depending on the sample configuration (**a**) for $0^{\circ }$ and (**b**) for $90^{\circ }$, the relative amounts of elements with local von Mises stress ${\bar{\sigma }}_{\mu }$ higher than the global one. The data involve the last frame of all simulations. ${\bar{\sigma }}_{max}$ is the von Mises stress value from the true curves (Figure 8).

**Figure 12:**
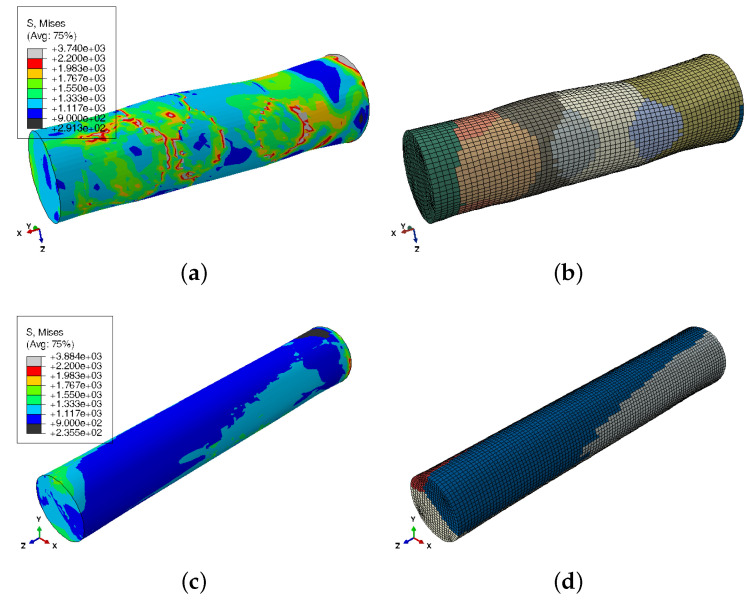
Contour plot (**a**,**c**) of the von Mises stress for the two different specimen configurations (**b**) = 90${}^{\circ }$ and (**d**) = 0${}^{\circ }$ at the last frame. Image on the right represents the deformed grain structure. Stresses are in MPa.

Finally, the stress concentration on grain boundaries was analysed. To that end, the last frame of all simulations was chosen. The maximum macroscopic von Mises ${\bar{\sigma }}_{max}$ obtained from the true stress–strain relationships, assuming an uniaxial stress state, was set as threshold and the amount of elements with a higher von Mises stress ${\bar{\sigma }}_{\mu }$ was calculated. Figure 11 collects these results. The specimens oriented perpendicularly to the grain-growth direction have around ten percent of the elements with stresses 1.2 times higher the macroscopic von Mises stress, and marginally, there are elements reaching values close to 2. However, the stress ratios ${\bar{\sigma }}_{\mu }/{\bar{\sigma }}_{max}$ for the 0${}^{\circ }$-sample elements are in the range of 1.0–1.2. Their highly oriented texture means that mechanical properties among grains are quite homogeneous and the stress concentration close to the crystal-crystal transition is rather small (see Figure 12c,d), unlike the 90${}^{\circ }$ specimens (see Figure 12a,b). The latter present quite different responses among grains, leading to von-Mises-stress ratios in the grain boundaries close to two. Hence, the simulation strategy is also able to predict the cavity nucleation dependence with the orientation in the case of DS alloys [7].

## 4. Conclusions

The flow stress of a DS nickel-base superalloy has been studied experimentally and numerically under different strain rates. The mechanical tests showed the strain-rate insensitivity behaviour of the alloy and an important anisotropy, including Young’s modulus, yielding point and hardening, depending on the orientation of the specimen: samples with columnar grain structures or samples obtained perpendicularly to the solidification direction.

The numerical strategy chosen, based on simulating the whole gauge length with a crystal plasticity model, was able to predict with high fidelity the experimental true stress–strain curves in the different directions. To that end, the information of quasi-static tests in the grain-growth direction was only used to fit the three constants of the hardening law while the yield strength at high-strain rates was used to establish the rate dependent parameter. Hence, the present model can be used as a tool for virtual testing and predicting mechanical properties in new directions. The response of each crystal was formulated with phenomenological laws, although the dislocation–dislocation interactions and junctions were taken into account. Going beyond the macrofields, the simulations displayed that the first stages of plastic deformation tend to be accommodated by the grains with higher Schmid factors, but when the test goes on, the rest of grains increase their importance. Lastly, the influence of the texture on the grain boundary stress concentrations is well predicted according to literature.

## Figures and Tables

**Table 1:** Chemical composition of MAR-M247 (wt.%).

Cr	Co	Al	Ti	W	Ta	Mo	C	Hf	Ni
8.00	10.0	5.50	1.00	10.0	3.00	0.60	0.15	1.5	Bal.

**Table 2:** Parameters of the CP model for MAR-M247. The elastic constants were obtained from [43], while the values that define the expression (10) were fitted from the experimental data in the parallel direction.

$C_{11}$	$C_{12}$	$C_{44}$	$h_{0}$	$\tau_{0}$	$\tau_{s}$	m
258.6 GPa	167.0 GPa	125.0 GPa	1.36$\tau_{0}$	$\tau_{0}$	2.26 $\tau_{0}$	0.0015
${\dot{\gamma }}_{0}$	$q_{1}$	$q_{2}$	$q_{3}$	$q_{4}$	$q_{5}$	$q_{6}$
0.001 s${}^{-1}$	1.00	1.00	5.38	0.68	1.12	0.96

## Abbreviations

The following abbreviations are used in this manuscript:

CP	Crystal plasticity
CPFEM	Crystal-plasticity-finite-element method
DS	Directionally solidified
FCC	Face centred cubic
SX	Single crystal
$T_{m}$	Melting temperature
VPSC	Visco plastic self consistent
SHTB	Split Hopkinson tension bar
fps	Frames per second
CRSS	Critical-resolved-shear stress
RVE	Representative volume element
C3D8	Eight-node fully-integrated hexahedral element
PDF	Probability density function
